# Estimating the Stature of an Ancient North Andean Population From Articular Breadths and Diaphyseal Diameters: An Extension of Anzellini and Toyne (2020)

**DOI:** 10.1002/ajpa.70310

**Published:** 2026-07-02

**Authors:** Armando Anzellini, J. Marla Toyne

**Affiliations:** ^1^ Department of Sociology and Anthropology Lehigh University Bethlehem Pennsylvania USA; ^2^ Department of Anthropology University of Central Florida Orlando Florida USA

**Keywords:** commingled remains, femur, ridge regression, tibia

## Abstract

Stature estimation of skeletal remains is mostly dependent on the lengths of long bones, but these are often unavailable in fragmented or commingled assemblages. To overcome this obstacle, we present an extension of the unisex stature estimation models for high‐altitude Andean populations presented by Anzellini and Toyne (2020) using femoral and tibial articular breadths and diaphyseal diameters. We estimated the anatomical statures of 28 adults, both males and females, from Kuelap, Peru, and created linear regression formulae using Ridge regression with k‐fold cross‐validation. Thirty models were generated, with over half achieving standard error values below ±4 cm and tibial multivariable models approaching the precision of length‐based estimates. These results demonstrate that epiphyseal breadths and midshaft diameters offer reliable alternatives for stature estimation when complete elements are unavailable, expanding options for analyses and providing valuable information that may otherwise go ignored.

## RESUMEN

La aproximación de la estatura de los restos óseos depende sobre todo de las medidas de longitud de huesos largos, pero estas medidas no suelen estar disponibles en contextos fragmentados o mezclados. Para resolver este dilema, presentamos una extensión de los modelos unisex de aproximación de estatura para poblaciones andinas presentados por Anzellini y Toyne (2020), utilizando anchuras articulares y diámetros diafisarios femorales y tibiales. Se estimaron las estaturas anatómicas de 28 adultos, tanto hombres como mujeres, del sitio de Kuelap, Peru, y se crearon fórmulas de regresión lineal utilizando la regresión Ridge con validación cruzada k‐fold. Se generaron 30 modelos, de los cuales más de la mitad alcanzaron valores de error estándar inferiores a ±4 cm y modelos multivariables tibiales que se aproximan a la precisión de las estimaciones basadas en la longitud. Estos resultados demuestran que las medidas de las articulaciones y los diámetros de diáfisis ofrecen alternativas fiables para la aproximación de la estatura cuando no se dispone de huesos largos completos, ampliando las opciones de análisis y proporcionando información valiosa que de otro modo podría ser ignorada.

Stature of a population can be used as a proxy for health, can be correlated with subsistence, and can help track secular changes in growth and nutritional status across time (Auerbach 2011; Byers 1994; Fogel et al. 1983). However, skeletal stature estimations are only effective when there are appropriate local/regional skeleton‐based estimation formulae that have been developed to account for population specific history, genetic, and geographic adaptations (e.g., Genovés (1967) for Mexico; Trotter and Gleser (1952) for US based “white” and “black” males; Anzellini and Toyne (2020) for high‐altitude northern Andean populations). In addition, archeological settings lacking contextual information and population‐based stature estimates have provided an opening for pseudoarchaeology and misinformation, which can only be countered with appropriate estimates (Landol 2024). The estimation of stature using published formulae, for both males and females, is most frequently based on those metrics that most account for the greatest variance in stature, namely the lengths of the femur and tibia. However, many contexts across the world suffer from taphonomic changes to long bones that include the fragmentation of the remains, the erosion of the epiphyses, or the commingling of fragmented remains, especially those contexts that disassociate diaphyses from epiphyses. In such cases, it may also be impossible or impractical to estimate sex, even from long bones (Anzellini and Toyne 2019), and most formulae for stature estimation are based on presumed sex (e.g., Trotter and Gleser 1952; Genovés 1967; Auerbach and Ruff 2010; Pomeroy and Stock 2012). Yet, such demographic data, even in those more difficult contexts, can provide valuable information that may be frequently ignored or remain unobserved (Osterholtz et al. 2014; Brickley and Buckberry 2015; Meyer et al. 2020; Judd 2020; Cao et al. 2024). Therefore, an approach that can explore demographic information, such as stature, beyond estimating the minimum number of individuals in those challenging contexts, can be extremely valuable in both bioarchaeological and forensic investigations (Garrido Varas and Intriago Leiva 2012; Gonçalves et al. 2014; Ubelaker 2014; Brickley and Buckberry 2015; Winbum et al. 2017; Anzellini and Toyne 2019; Judd 2020; Meyer et al. 2020).

Anzellini and Toyne (2020) demonstrated that, in commingled contexts, the particular problem of sex‐based approaches can be solved using unisex stature estimation formulae. They presented their results for high‐altitude north central Andean populations, demonstrating that unisex stature estimation was viable and reliable. The formulae presented in Anzellini and Toyne (2020) were based on anatomical stature estimates (Raxter et al. 2007; Fully 1956) from individuals recovered at the site of Kuelap, located in the high altitude rainforests of the northern Peruvian Andes. They demonstrated that unisex formulae for this population were reliable, but as expected, males were generally underestimated and females were overestimated when compared to anatomical stature estimates. As with other studies, however, that study only presented estimation formulae based on the lengths of each skeletal element of the lower limb.

With skeletal collections that preserve the ability of measuring long bone lengths, stature estimates for most reliable formulae fall within a standard error of less than 3.5 cm, or approximately 3% of the estimated stature (Genovés 1967; Auerbach and Ruff 2010; Pomeroy and Stock 2012; Anzellini and Toyne 2020). This standard error of approximately 3% is generally considered appropriate for most applications, including bioarchaeological and forensic analyses. In contexts with poor preservation of the epiphyses and metaphyses, or fragmentation of the remains, stature estimation formulae based on long bone lengths are not applicable. In these situations, less precise stature estimates, that still fall within a reasonable error (i.e., ±4.5 cm or approximately 4% error; Anzellini and Toyne (2020)), may still provide necessary and useful data, even those relying on less consistent metrics, such as diaphyseal diameters and articular breadths. Studies on body mass estimates have demonstrated the correlations between these long bone cross‐sectional and articular metrics and estimations of body mass (Ruff and Wood 2023; Wheeler et al. 2015; Calder 1996; Ruff 1990). In general, there is a positive correlation between stature and body mass (Ruff et al. 2012), but it must be noted that population histories and residential latitude and altitude will have a greater effect on body breath/mass than stature (Ruff 2002). For this reason, as well as for those outlined in Anzellini and Toyne (2020), population specific formulae are imperative for the application of nonlength metrics to the estimation of stature.

**FIGURE 1:**
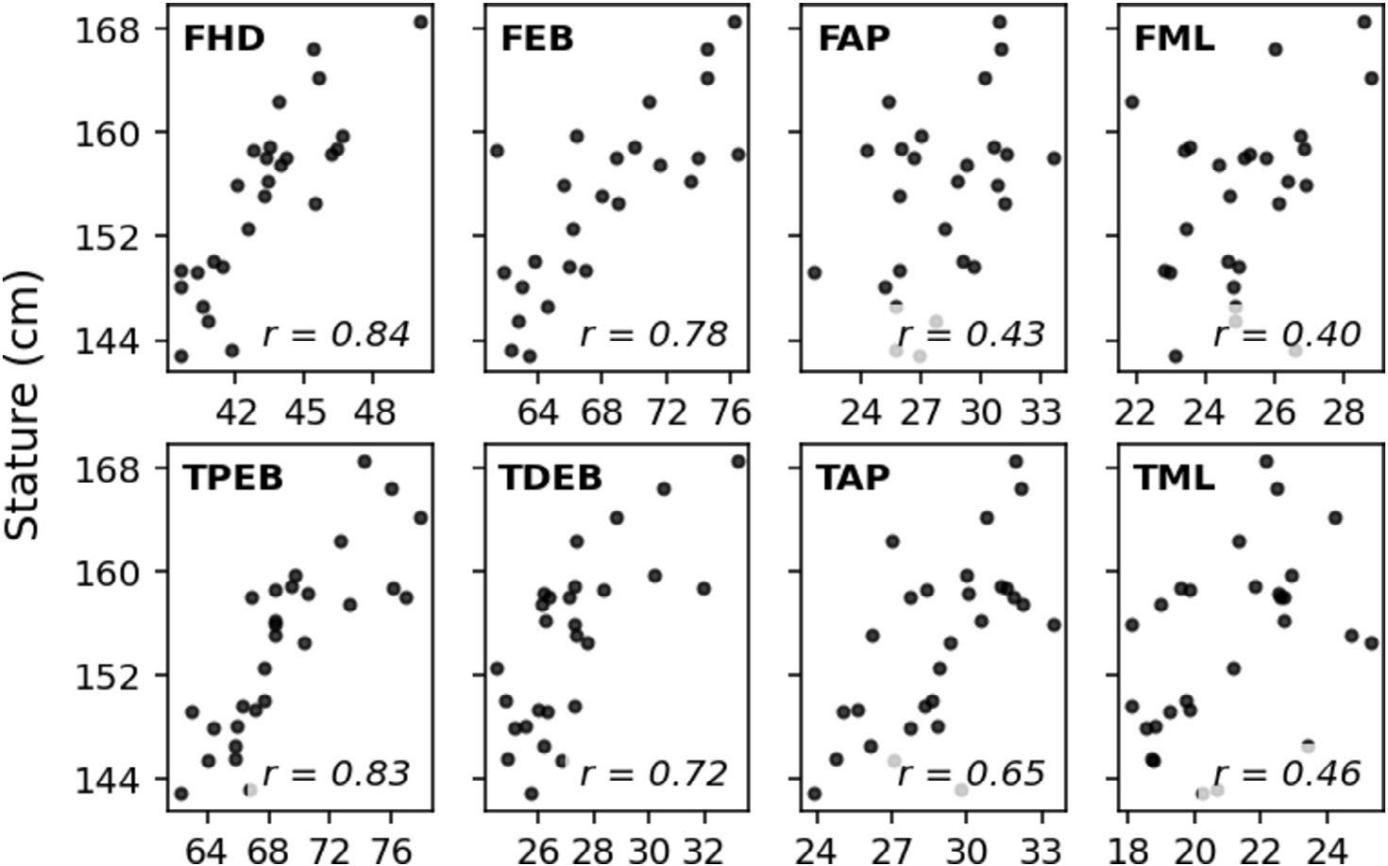
Correlations of long bone, nonlength metrics to stature. Pearson correlation coefficients (*r*) are given for each relationship. Definitions for abbreviations can be found in Table 1.

For these reasons, this technical note presents formulae for the estimation of stature from femoral and tibial diaphyseal diameters and articular breadths. As demonstrated by Anzellini and Toyne (2020), the greatest variation in population stature is explained by lengths; however, breadths and widths of long bones, especially those of the lower limbs, still correlate to variations in stature (Figure 1). From Figure 1, it is clear that articular breadths have a strong correlation to estimated stature, while some diaphyseal metrics, especially those of the tibia, have moderate correlations (abbreviations of metrics are presented in Table 1). This study is not the first to estimate stature from osteological metrics other than long bone lengths (Jantz and Ousley 2013; Chiba et al. 2018), but population‐specific estimation formulae based on articular breadths and diaphyseal diameters have not yet been created for a northern Andean population.

**TABLE 1:** Glossary of abbreviation for each of the metrics explored in this study.

Abbreviation	Measurement
FHD	Maximum Diameter of the Femoral Head
FEB	Epicondylar Breadth of the Femur
FAP	Anterio‐posterior Midshaft Diameter of the Femur
FML	Medio‐lateral Midshaft Diameter of the Femur
TPEB	Proximal Epiphyseal Breadth of the Tibia
TDEB	Distal Epiphyseal Breadth of the Tibia
TAP	Anterio‐posterior Midshaft Diameter of the Tibia
TML	Medio‐lateral Midshaft Diameter of the Tibia.

## 1 Materials and Methods

As with the original study, statures of complete and mostly complete individuals—the same sample population as the original study—were estimated using the Fully anatomical skeletal stature technique (Raxter et al. 2007; Fully 1956). The sex of complete individuals was estimated using characteristics of the *ossa coxae* and crania, and diaphyseal diameters and epiphyseal breadths of the tibia and femur were measured following Buikstra and Ubelaker (1994). The reference population used for this study consisted of young adults (20–35 years; *n* = 10), mature adults (35–50 years; *n* = 17), and one older adult (50+ years; *n* = 1) and included estimated males (*n* = 16) and estimated females (*n* = 12).

We took two approaches to avoid issues of multicollinearity in our formulae since long bone metrics of the same element are correlated: (1) we calculated the variance inflation factor (VIF) for all possible metric combinations to explore the significance of multicollinearity (Salmerón Gómez et al. 2018; Salmerón Gómez et al. 2016) and (2) we employed a Ridge regression which includes an L_2_ penalty term (*λ*) that reduces co‐linear coefficients to mitigate the effects of existing multicollinearity (Hoerl and Kennard 1970; Marquardt and Snee 1975; Herawati et al. 2024; O'Brien 2007). All estimated VIF values were between 1 and 4 for both femoral (Table 2) and tibial (Table 3) metrics, suggesting either low to moderate multicollinearity, but not significant in any of our cases. Any moderate collinearity observable between our variables is effectively mitigated with the Ridge regression method (O'Brien 2007).

**TABLE 2:** Variance inflation factor (VIF) for each of the metrics presented in each of the possible combinations. Rows represent each metric as a potential variable; columns represent the possible combination of metrics in each formula.

Variable	FHD	FEB	FEB + FAP	FEB + FML	FHD + FEB	FAP + FML	FHD + FAP	FHD + FML	FEB + FAP + FML	FHD + FEB + FAP	FHD + FEB + FML	FHD + FAP + FML	FHD + FEB + FAP + FML
FHD	1.00				2.45		1.30	1.63		2.45	3.12	1.75	3.19
FEB		1.00	1.89	1.24	2.45				1.92	3.27	2.46		3.48
FAP			1.89			1.30	1.30		2.16	1.89		1.40	2.22
FML				1.24		1.30		1.63	1.41		1.57	1.75	1.84

**TABLE 3:** Variance inflation factor (VIF) for each of the metrics presented in each of the possible combinations. Rows represent each metric as a potential variable, columns represent the possible combination of metrics in each formula.

Variable	TPEB	TDEB	TAP	TDEB + TAP	TDEB + TML	TPEB + TDEB	TAP + TML	TPEB + TAP	TPEB + TML	TDEB + TAP + TML	TPEB + TDEB + TAP	TPEB + TDEB + TML	TPEB + TAP + TML	TPEB + TDEB + TAP + TML
TPEB	1.00					1.58		2.04	1.22		2.58	1.76	2.61	3.11
TDEB		1.00		1.31	1.09	1.58				1.40	1.59	1.58		1.59
TAP			1.00	1.31			1.02	2.04		1.31	2.14		2.19	2.31
TML					1.09		1.02		1.22	1.09		1.22	1.30	1.32

Using the Ridge regression process in the scikit‐learn Python package (Pedregosa et al. 2011), unisex formulae were created for the undifferentiated population for all available combinations of the metrics explored for each individual element. Formulae for isolated metrics were also calculated only if the correlation coefficient between that metric and stature was above 0.5 (Figure 1), since any correlation below that would be unreliable at best and be too dependent on the reference population, leading to overfitting. To validate the model and calculate the most effective *λ* for each regression (Table 4), we used a k‐fold cross validation with seven groups, each consisting of four individuals. Formulae were created using all diameters and articular breadths available for each element and their precision was evaluated using *R* ^2^ and the Standard Error of the Estimate (SEE) (Table 4). To explore the significance of the effect presented by each variable in each formula, the local Cohen's *f* ^2^ for effect size (Cohen 1988, 413; Selya et al. 2012) was calculated (Table 5). These values show that in all cases, articular breadths for both the femur and tibia had strong significant effects (*f* ^2^ > 0.35) or moderate effects (*f* ^2^ > 0.15) on the regression formulae. Tibial diaphyseal metrics showed mostly weak to moderate effects, although in some cases they demonstrated strong effects, when in conjunction with TPEB (Table 5). Femoral diaphyseal metrics, on the other hand, had weak (*f* ^2^ < 0.15) to very weak (*f* ^2^ ~ 0) effect sizes in all formulae (Table 5). This further reinforces what has been presented previously, namely that tibial metrics have a much stronger correlation to stature than femoral metrics (Anzellini and Toyne 2020).

**TABLE 4:** Linear regression formulae for stature estimation using long bone length diameters and articular breadths derived from the Kuelap data including their respective standard error of estimate (SEE) and *R* ^2^ values.

Variables	Formula	SEE	*R* ^2^	*λ*
TPEB + TDEB + TAP + TML	60.823 + (0.715 * TPEB) + (0.739 * TDEB) + (0.515 * TAP) + (0.452 * TML)	2.97	0.81	37.93
TPEB + TDEB + TML	61.33 + (0.926 * TPEB) + (0.793 * TDEB) + (0.37 * TML)	3.16	0.79	37.93
TPEB + TDEB + TAP	64.08 + (0.825 * TPEB) + (0.769 * TDEB) + (0.438 * TAP)	3.16	0.79	37.93
TPEB + TDEB	64.011 + (0.992 * TPEB) + (0.812 * TDEB)	3.27	0.77	37.93
TDEB + TAP + TML	68.389 + (1.241 * TDEB) + (1.179 * TAP) + (0.88 * TML)	3.30	0.77	8.86
FHD + FEB + FAP + FML	58.946 + (1.644 * FHD) + (0.612 * FEB) + (−0.278 * FAP) + (−0.352 * FML)	3.34	0.78	8.86
FHD + FEB + FML	59.991 + (1.678 * FHD) + (0.508 * FEB) + (−0.481 * FML)	3.39	0.77	8.86
FHD + FEB + FAP	55.97 + (1.513 * FHD) + (0.643 * FEB) + (−0.36 * FAP)	3.40	0.77	8.86
FHD + FEB	53.488 + (1.624 * FHD) + (0.461 * FEB)	3.50	0.76	2.07
FHD + FML	64.713 + (2.505 * FHD) + (−0.714 * FML)	3.59	0.74	2.07
FHD + FAP + FML	66.536 + (2.258 * FHD) + (0.212 * FAP) + (−0.599 * FML)	3.59	0.74	8.86
FHD	58.524 + (2.233 * FHD)	3.75	0.71	0.48
FHD + FAP	62.291 + (2.066 * FHD) + (0.123 * FAP)	3.76	0.71	8.86
TPEB + TAP + TML	63.499 + (0.986 * TPEB) + (0.447 * TAP) + (0.466 * TML)	3.80	0.71	37.93
TDEB + TAP	80.376 + (1.473 * TDEB) + (1.187 * TAP)	3.82	0.69	8.86
TPEB + TML	59.542 + (1.238 * TPEB) + (0.441 * TML)	3.88	0.70	8.86
TPEB + TAP	62.7 + (1.182 * TPEB) + (0.345 * TAP)	3.94	0.69	8.86
TPEB	60.924 + (1.352 * TPEB)	3.98	0.68	0.00
TDEB + TML	81.197 + (1.993 * TDEB) + (0.915 * TML)	4.33	0.60	2.07
FEB + FAP + FML	79.179 + (1.094 * FEB) + (−0.204 * FAP) + (0.275 * FML)	4.40	0.62	37.93
FEB + FAP	83.176 + (1.113 * FEB) + (−0.148 * FAP)	4.45	0.61	37.93
FEB + FML	79.619 + (1.026 * FEB) + (0.214 * FML)	4.47	0.60	37.93
FEB	82.824 + (1.057 * FEB)	4.49	0.60	37.93
TAP + TML	81.83 + (1.606 * TAP) + (1.241 * TML)	4.67	0.56	2.07
TDEB	96.71 + (2.131 * TDEB)	4.75	0.52	8.86

**TABLE 5:** Local Cohen's *f* ^2^ values of effect size of each metric as a variable. Columns represent each metric as a potential variable, rows represent the combination of metrics in each formula.

	FHD	FEB	FAP	FML	TPEB	TDEB	TAP	TML
FEB + FML	0.74		0.05					
FEB + FAP + FML	0.73							
FEB + FAP	0.70			0.03				
FEB	0.67		0.03	0.00				
FHD + FAP		0.26		0.12				
FHD		0.21	0.00	0.12				
FHD + FAP + FML		0.18						
FHD + FML		0.13	0.00					
FHD + FEB + FML			0.05					
FHD + FEB			0.04	0.04				
FHD + FEB + FAP				0.05				
TDEB					1.09		0.55	0.20
TDEB + TML					0.90		0.74	
TAP + TML					0.52	0.91		
TDEB + TAP					0.48			0.35
TDEB + TAP + TML					0.21			
TPEB + TAP + TML						0.53		
TPEB + TAP						0.48		0.07
TPEB + TML						0.43	0.03	
TPEB						0.39	0.03	0.07
TPEB + TDEB + TML							0.11	
TPEB + TDEB							0.10	0.10
TPEB + TDEB + TAP								0.11

## 2 Findings

As seen in Table 4, at least three of the formulae, specifically multivariable stature estimates of the tibia, show SEE values that approximate or are below the SEE value for the formula using the condylo‐malleolar tibial length (SEE = 3.117; Anzellini and Toyne (2020)). Estimates for males using these formulae were much closer to the anatomical estimates than for females, this likely being the result of the slight skew in the sample demographics (see Supporting Information). Others, however, show significantly higher SEE values, with the worse models approaching ±4.75 cm. As a result, we suggest not using any formula presented in the table with an *R* ^2^ below 0.65, or an SEE above 4 cm, as these suggest a weak relationship between the metrics and stature and a percentage of error that is higher than reasonably expected for most research questions. Yet more than half of the linear models (18 of the 25 formulae) have SEE values below ±4 cm, which is more than adequate for bioarchaeological analyses. Results also demonstrate that dimensions of the tibia tend to produce the most accurate estimates when compared to the anatomical stature estimate and that those formulae providing a more complete geometry of the skeletal element (i.e., those that use more than one metric) are more reliable than single‐metric formulae. For those formulae that have large standard errors when compared to those created using maximum and physiological lengths, even a range of 9.5 cm (i.e., ±4.75 cm) may still be better than not having a stature estimate at all, depending on the research question being posed. The formulae presented here, while generally less precise than those created using long bone lengths (Anzellini and Toyne 2020), are still useful and can approximate the precisions observed using the standard estimates based on long bone lengths. In situations where lengths are not available, and with complete transparency, these stature estimates based on articular breadths and midshaft diameters can provide usable, key results that were previously considered inaccessible. These additional data enrich commingled and isolated contexts and can be integrated into larger questions of population specific health factors (Brickley and Buckberry 2015). In the future, researchers may follow the approach presented here to create population specific formulae for stature estimation from epiphyseal breadths and midshaft diameters for other ecogeographic populations.

## Author Contributions

**J. Marla Toyne:** data curation, writing – review and editing, validation. **Armando Anzellini:** conceptualization, methodology, software, writing – original draft, writing – review and editing.

## Ethics Statement

All analyses of skeletal remains were conducted with the consent, collaboration, and assistance of the local community of Kuelap, the Peruvian Ministerio de Cultura, and the Dirección Desconcentrada de Cultura (DDC) de Amazonas.

## Supporting information


**Table S1:** Individuals analyzed including their estimated sex and age, relevant metrics, anatomical stature estimate and the estimates obtained using the formulae presented in the article (1 to 5).
**Figure S1:** Mean deviation of stature estimates from Anatomical Stature by formula presented in the study. Lowest deviations on the left and higher deviations on the right.

## Data Availability

The data that supports the findings of this study are available in the Supporting Information of this article.
