# Supplementary material for: Estimating the Stature of an Ancient North Andean Population From Articular Breadths and Diaphyseal Diameters: An Extension of Anzellini and Toyne (2020)
Source: Am J Biol Anthropol. 2026 Jul 2;190(3):e70310. doi: 10.1002/ajpa.70310 (PMC13329083; doi:10.1002/ajpa.70310)

Supplementary Table. Individuals analyzed including their estimated sex and age, relevant metrics, anatomical stature estimate and the estimates obtained using the formulae presented in the article. 1 of 5

Individual ID	Estimated Sex	Age Category	Side	Osteometrics							
				FHD	FEB	FAP	FML	TPEB	TDEB	TAP	TML
KCAc1-IIM ENT1	F	YA	L	40.27	61.91	21.63	22.94	63.02	26.32	25.07	19.26
KCPlatII -IIIÑ Ent3A	F	MA	L	43.30	68.90	26.60	25.10	66.90	27.10	27.70	22.70
K-PAC Est2 -IIN' Ent3b	F	MA	L					65.83	24.91	24.74	18.71
K-PAC Est2 -IIN' Ent1a	F	YA	L	39.59	63.44	26.89	23.12	62.22	25.73	23.89	20.22
K-PAC Est2 -IIN' Ent1b	F	MA	R	40.55	64.52	25.71	24.86	65.84	26.19	26.15	23.39
K-PAC Est2 -IIN' Ent1c	F	MA	L	39.60	66.90	25.89	22.79	67.11	25.98	25.61	19.85
K-PAC Est2 -IIN' Ent2A	F	MA	R	41.40	65.91	29.65	24.94	66.29	27.32	28.33	18.08
K-PAS MO -VIII U' ENT45	F	YA	L					64.41	25.15	27.76	18.55
K-PAS MO -VIII U' ENT63	F	MA	R	40.99	63.79	29.11	24.63	67.75	24.83	28.60	19.71
KSPlatC E4-VIa ENT65	F	YA	L	39.57	62.99	25.22	24.78	65.95	25.56	28.83	18.78
KSPlatC E6-VIIIz-VIIIa' ENT85	F	OA	L	41.84	62.35	25.73	26.59	66.73		29.79	20.69
K-TM E38 -VIII V Ent1	F	MA	L	40.77	62.73	27.71	24.83	64.05	26.85	27.08	18.75
K-TM E33 -IX W Ent1	M	MA	L	50.03	76.22	30.95	28.58	74.23	33.19	31.93	22.16
K-PAC Est2 -IIN' Ent3a	M	YA	R	45.33	74.47	31.04	26.00	75.94	30.51	32.16	22.47
K-SbPl II -VIII S E1 Ent1b	M	MA	L	43.48	69.99	30.67	23.56	69.53	27.29	31.39	21.84
K-PAC E1 -III G' Ent1	M	MA	R	44.14	74.00	33.69	25.75	76.98	26.37	31.88	22.57
KSPlatC E4-VIa ENT57	M	MA	L	43.97	71.62	29.30	24.38	73.25	26.13	32.26	18.97
KSPlatC E6-VIIIz-VIIIa' ENT84	M	MA	L	42.05	65.57	30.82	26.91	68.45	27.34	33.49	18.09
KSPlatC E6-VIIIz-VIIIa' ENT81	M	YA	L	46.65	66.39	26.97	26.73	69.72	30.22	29.96	22.88
KSPlatC E6-VIIz-VIIa'-VIIIz ENT80	M	MA	L	46.38		25.98	26.87	76.14	31.94	31.56	19.58
KSTIN -IIU Ent12	M	MA	L	45.60	74.50	30.20	28.80	77.90	28.80	30.80	24.20
KSPlatC E4-VIa ENT66	M	MA	L	42.55	66.20	28.18	23.42	67.65	24.52	28.86	21.18
KSPlatC Patio-VIz ENT1A	M	YA	L	42.75	61.46	24.29	23.38	68.45	28.34	28.40	19.85
KSSbPlt1 -IOE8 Ent2	M	YA	L	43.20	68.00	25.90	24.70	68.40	27.40	26.20	24.70
KSPlat2 E8 -IIIR ENT1	M	MA	R	43.38	73.48	28.84	26.40	68.37	26.26	30.57	22.68
K-PAC Est2 -IIN' Ent5a	M	YA	L	46.15	76.41	31.29	25.27	70.50	26.20	30.06	22.50
K-SbPltII -VI U Ent1	M	YA	R	43.89	70.94	25.33	21.85	72.71	27.35	27.00	21.30
K-SbPltII -VI U Ent2	M	MA	R	45.41	69.03	31.19	26.10	70.33	27.79	29.34	25.32

Supplementary Table. Individuals analyzed including their estimated sex and age, relevant metrics, anatomical stature estimate and the estimates obtained using the formulae presented in the article.

Individual ID	Anatomical Stature	Estimates from Formulae			
		TPEB + TDEB + TAP + TML	TPEB + TDEB + TML	TPEB + TDEB + TAP	TPEB + TDEB
KCAc1-IIM ENT1	149.2	146.9	147.7	147.3	147.9
KCPlatII -IIIÑ Ent3A	158.0	153.2	153.2	152.2	152.4
K-PAC Est2 -IIN' Ent3b	145.6	147.5	149.0	148.4	149.5
K-PAC Est2 -IIN' Ent1a	142.9	145.8	146.8	145.7	146.6
K-PAC Est2 -IIN' Ent1b	146.6	151.3	151.7	150.0	150.6
K-PAC Est2 -IIN' Ent1c	149.4	150.2	151.4	150.6	151.7
K-PAC Est2 -IIN' Ent2A	149.7	151.2	151.1	152.2	152.0
K-PAS MO -VIII U' ENT45	148.0	148.1	147.8	148.7	148.3
K-PAS MO -VIII U' ENT63	150.0	151.3	151.0	151.6	151.4
KSPlatC E4-VIa ENT65	148.1	150.2	149.6	150.8	150.2
KSPlatC E6-VIIIz-VIIIa' ENT85	143.2				
K-TM E38 -VIII V Ent1	145.4	148.9	148.9	149.4	149.4
K-TM E33 -IX W Ent1	168.5	164.9	164.6	164.8	164.6
K-PAC Est2 -IIN' Ent3a	166.4	164.4	164.2	164.3	164.1
K-SbPl II -VIII S E1 Ent1b	158.9	156.7	155.4	156.2	155.1
K-PAC E1 -III G' Ent1	158.1	162.0	161.9	161.8	161.8
KSPlatC E4-VIa ENT57	157.6	157.7	156.9	158.7	157.9
KSPlatC E6-VIIIz-VIIIa' ENT84	156.0	155.4	153.1	156.2	154.1
KSPlatC E6-VIIIz-VIIIa' ENT81	159.7	158.8	158.3	158.0	157.7
KSPlatC E6-VIIz-VIIa'-VIIIz ENT80	158.8	164.0	164.4	165.3	165.5
KSTIN -IIU Ent12	164.3	164.6	165.3	164.0	164.7
KSPlatC E4-VIa ENT66	152.5	151.7	151.3	151.4	151.0
KSPlatC Patio-VIz ENT1A	158.6	154.3	154.5	154.8	154.9
KSSbPlt1 -IOE8 Ent2	155.1	154.6	155.5	153.1	154.1
KSPlat2 E8 -IIIR ENT1	156.2	155.1	153.9	154.1	153.2
K-PAC Est2 -IIN' Ent5a	158.3	156.2	155.7	155.6	155.2
K-SbPltII -VI U Ent1	162.4	156.6	158.2	156.9	158.3
K-SbPltII -VI U Ent2	154.6	158.2	157.9	156.3	156.3

Supplementary Table. Individuals analyzed including their estimated sex and age, relevant metrics, anatomical stature estimate and the estimates obtained using the formulae presented in the article. 3 of 5

	Estimates from Formulae					
Individual ID	TDEB + TAP + TML	FHD + FEB + FAP + FML	FHD + FEB + FML	FHD + FEB + FAP	FHD + FEB	FHD + FML
KCAc1-IIM ENT1	147.6	149.0	148.0	148.9	147.4	149.2
KCPlatII -IIIÑ Ent3A	154.7	156.1	155.6	156.2	155.6	155.3
K-PAC Est2 -IIN' Ent3b	144.9					
K-PAC Est2 -IIN' Ent1a	146.3	147.2	147.5	147.0	147.0	147.4
K-PAC Est2 -IIN' Ent1b	152.3	149.2	148.9	149.6	149.1	148.5
K-PAC Est2 -IIN' Ent1c	148.3	149.8	149.5	149.6	148.6	147.6
K-PAC Est2 -IIN' Ent2A	151.6	150.3	150.9	150.3	151.1	150.6
K-PAS MO -VIII U' ENT45	148.7					
K-PAS MO -VIII U' ENT63	150.3	148.6	149.3	148.5	149.5	149.8
KSPlatC E4-VIa ENT65	150.6	146.8	146.5	147.3	146.8	146.1
KSPlatC E6-VIIIz-VIIIa' ENT85		149.4	149.1	150.1	150.2	150.5
K-TM E38 -VIII V Ent1	150.1	147.9	148.3	148.0	148.6	149.1
K-TM E33 -IX W Ent1	166.7	169.2	168.9	169.5	169.9	169.6
K-PAC Est2 -IIN' Ent3a	163.9	161.3	161.4	161.3	161.4	159.7
K-SbPl II -VIII S E1 Ent1b	158.5	156.4	157.2	155.7	156.4	156.8
K-PAC E1 -III G' Ent1	158.6	158.4	159.3	158.2	159.3	156.9
KSPlatC E4-VIa ENT57	155.5	158.3	158.4	158.0	157.9	157.5
KSPlatC E6-VIIIz-VIIIa' ENT84	157.7	150.2	150.9	150.7	152.0	150.8
KSPlatC E6-VIIIz-VIIIa' ENT81	161.3	159.4	159.1	159.5	159.9	162.5
KSPlatC E6-VIIz-VIIa'-VIIIz ENT80	162.5					161.7
KSTIN -IIU Ent12	161.7	161.0	160.5	162.0	161.9	158.4
KSPlatC E4-VIa ENT66	151.5	153.3	153.8	152.8	153.1	154.6
KSPlatC Patio-VIz ENT1A	154.5	151.9	151.7	151.4	151.2	155.1
KSSbPlt1 -IOE8 Ent2	155.0	155.7	155.1	155.7	155.0	155.3
KSPlat2 E8 -IIIR ENT1	157.0	157.9	157.4	158.5	157.8	154.5
K-PAC Est2 -IIN' Ent5a	156.1	164.0	164.1	163.7	163.7	162.3
K-SbPltII -VI U Ent1	152.9	159.8	159.2	158.9	157.5	159.1
K-SbPltII -VI U Ent2	159.7	158.0	158.7	157.8	159.1	159.8

Supplementary Table. Individuals analyzed including their estimated sex and age, relevant metrics, anatomical stature estimate and the estimates obtained using the formulae presented in the article.

	Estimates from Formulae							
Individual ID	FHD + FAP + FML	FHD	FHD + FAP	TPEB + TAP + TML	TDEB + TAP	TPEB + TML	TPEB + TAP	TPEB
KCAc1-IIM ENT1	148.3	148.4	148.1	145.8	148.9	146.1	145.8	146.1
KCPlatII -IIIÑ Ent3A	154.9	155.2	155.0	152.4	153.2	152.4	151.3	151.4
K-PAC Est2 -IIN' Ent3b				148.2	146.4	149.3	149.0	149.9
K-PAC Est2 -IIN' Ent1a	147.8	146.9	147.4	144.9	146.6	145.5	144.5	145.0
K-PAC Est2 -IIN' Ent1b	148.7	149.1	149.2	151.0	150.0	151.4	149.5	149.9
K-PAC Est2 -IIN' Ent1c	147.8	147.0	147.3	150.4	149.0	151.4	150.9	151.7
K-PAC Est2 -IIN' Ent2A	151.4	151.0	151.5	149.9	154.2	149.6	150.8	150.5
K-PAS MO -VIII U' ENT45				148.1	150.4	147.5	148.4	148.0
K-PAS MO -VIII U' ENT63	150.5	150.1	150.6	152.3	150.9	152.1	152.6	152.5
KSPlatC E4-VIa ENT65	146.4	146.9	147.1	150.2	152.2	149.5	150.6	150.1
KSPlatC E6-VIIIz-VIIIa' ENT85	150.5	152.0	151.9	152.3		151.3	151.9	151.1
K-TM E38 -VIII V Ent1	149.6	149.6	149.9	147.5	152.1	147.1	147.7	147.5
K-TM E33 -IX W Ent1	168.9	170.2	169.5	161.3	167.2	161.2	161.5	161.3
K-PAC Est2 -IIN' Ent3a	159.9	159.7	159.8	163.2	163.5	163.5	163.6	163.6
K-SbPl II -VIII S E1 Ent1b	157.1	155.6	155.9	156.3	157.8	155.3	155.7	154.9
K-PAC E1 -III G' Ent1	157.9	157.1	157.6	164.2	157.1	164.8	164.7	165.0
KSPlatC E4-VIa ENT57	157.4	156.7	156.7	159.0	157.2	158.6	160.4	160.0
KSPlatC E6-VIIIz-VIIIa' ENT84	151.9	152.4	153.0	154.4	160.4	152.3	155.2	153.5
KSPlatC E6-VIIIz-VIIIa' ENT81	161.6	162.7	162.0	156.3	160.5	155.9	155.4	155.2
KSPlatC E6-VIIz-VIIa'-VIIIz ENT80	160.7	162.1	161.3	161.8	164.9	162.4	163.6	163.9
KSTIN -IIU Ent12	158.7	160.3	160.2	165.4	159.4	166.7	165.4	166.2
KSPlatC E4-VIa ENT66	154.6	153.5	153.7	153.0	150.8	152.6	152.6	152.4
KSPlatC Patio-VIz ENT1A	154.2	154.0	153.6	152.9	155.8	153.0	153.4	153.5
KSSbPlt1 -IOE8 Ent2	154.8	155.0	154.7	154.2	151.8	155.1	152.6	153.4
KSPlat2 E8 -IIIR ENT1	154.8	155.4	155.5	155.1	155.3	154.2	154.1	153.4
K-PAC Est2 -IIN' Ent5a	162.2	161.6	161.5	156.9	154.6	156.7	156.4	156.2
K-SbPltII -VI U Ent1	157.9	156.5	156.1	157.2	152.7	159.0	158.0	159.2
K-SbPltII -VI U Ent2	160.1	159.9	159.9	157.8	156.1	157.8	156.0	156.0

Supplementary Table. Individuals analyzed including their estimated sex and age, relevant metrics, anatomical stature estimate and the estimates obtained using the formulae presented in the article. 5 of 5

	Estimates from Formulae						
Individual ID	TDEB + TML	FEB + FAP + FML	FEB + FAP	FEB + FML	FEB	TAP + TML	TDEB
KCAc1-IIM ENT1	151.3	148.8	148.9	148.0	148.3	146.0	152.8
KCPlatII -IIIÑ Ent3A	156.0	156.0	155.9	155.7	155.7	154.5	154.5
K-PAC Est2 -IIN' Ent3b	148.0					144.8	149.8
K-PAC Est2 -IIN' Ent1a	151.0	149.5	149.8	149.7	149.9	145.3	151.5
K-PAC Est2 -IIN' Ent1b	154.8	151.4	151.2	151.1	151.0	152.9	152.5
K-PAC Est2 -IIN' Ent1c	151.1	153.4	153.8	153.1	153.5	147.6	152.1
K-PAC Est2 -IIN' Ent2A	152.2	152.1	152.1	152.6	152.5	149.8	154.9
K-PAS MO -VIII U' ENT45	148.3					149.4	150.3
K-PAS MO -VIII U' ENT63	148.7	149.8	149.9	150.3	150.3	152.2	149.6
KSPlatC E4-VIa ENT65	149.3	149.8	149.6	149.5	149.4	151.4	151.2
KSPlatC E6-VIIIz-VIIIa' ENT85		149.5	148.8	149.3	148.7	155.3	
K-TM E38 -VIII V Ent1	151.9	149.0	148.9	149.3	149.1	148.6	153.9
K-TM E33 -IX W Ent1	167.6	164.1	163.4	163.9	163.4	160.6	167.4
K-PAC Est2 -IIN' Ent3a	162.6	161.5	161.5	161.6	161.5	161.4	161.7
K-SbPl II -VIII S E1 Ent1b	155.6	156.0	156.5	156.5	156.8	159.3	154.9
K-PAC E1 -III G' Ent1	154.4	160.3	160.6	161.1	161.0	161.0	152.9
KSPlatC E4-VIa ENT57	150.6	158.3	158.6	158.3	158.5	157.2	152.4
KSPlatC E6-VIIIz-VIIIa' ENT84	152.2	152.0	151.6	152.7	152.1	158.1	155.0
KSPlatC E6-VIIIz-VIIIa' ENT81	162.4	153.7	153.1	153.5	153.0	158.3	161.1
KSPlatC E6-VIIz-VIIa'-VIIIz ENT80	162.8					156.8	164.8
KSTIN -IIU Ent12	160.7	162.4	161.6	162.2	161.6	161.3	158.1
KSPlatC E4-VIa ENT66	149.4	152.3	152.7	152.6	152.8	154.5	149.0
KSPlatC Patio-VIz ENT1A	155.8	147.9	148.0	147.7	147.8	152.1	157.1
KSSbPlt1 -IOE8 Ent2	158.4	155.1	155.0	154.7	154.7	154.6	155.1
KSPlat2 E8 -IIIR ENT1	154.3	160.9	160.7	160.7	160.5	159.1	152.7
K-PAC Est2 -IIN' Ent5a	154.0	163.3	163.6	163.4	163.6	158.0	152.5
K-SbPltII -VI U Ent1	155.2	157.6	158.4	157.1	157.8	151.6	155.0
K-SbPltII -VI U Ent2	159.8	155.5	155.4	156.0	155.8	160.4	155.9

Supplementary Figure. Mean deviation of stature estimates from Anatomical Stature by formula presented in the study. Lowest deviations on the left and higher deviations on the right

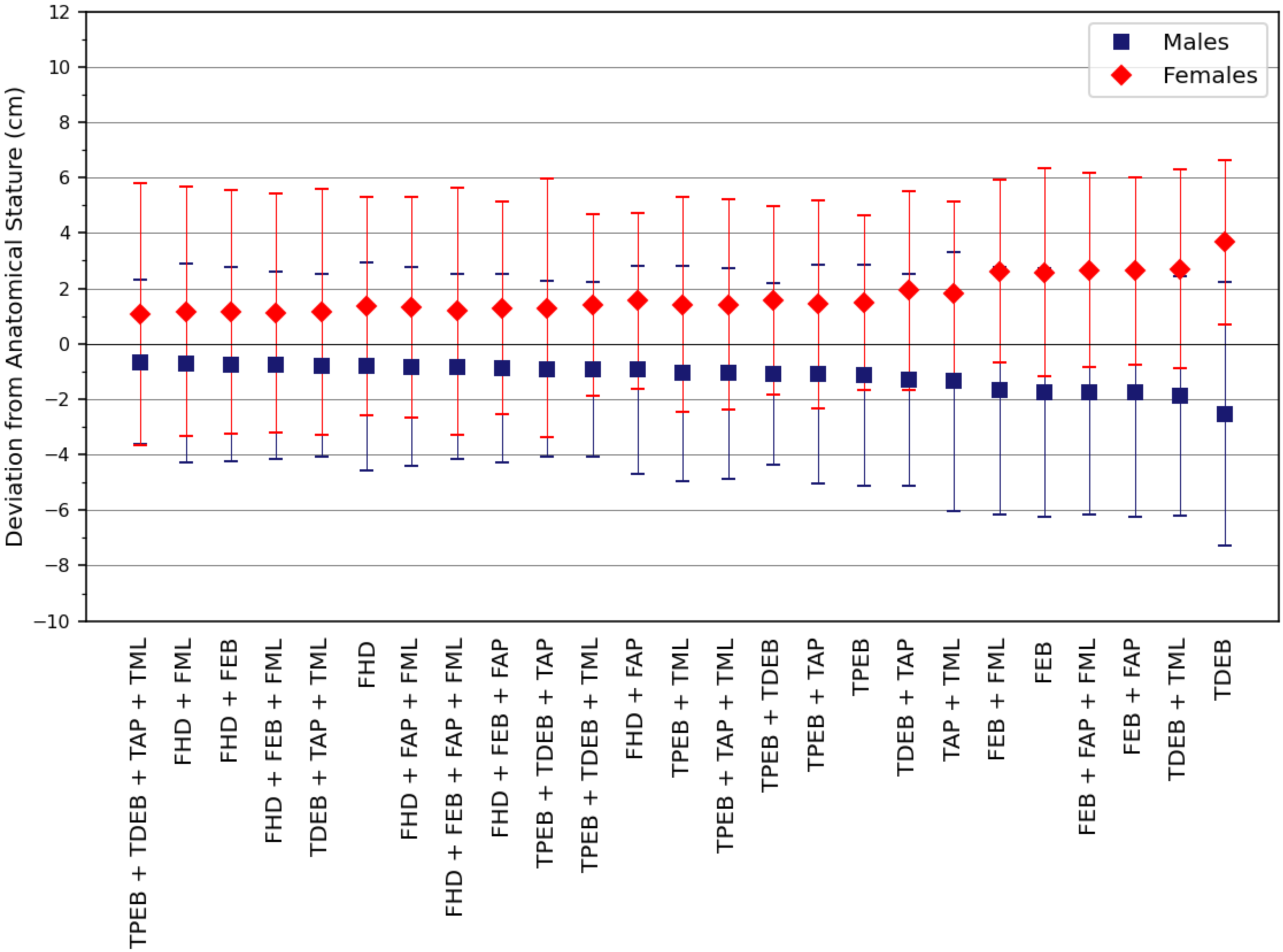

Supplement: Supplementary file 1 — Table S1: Individuals analyzed including their estimated sex and age, relevant metrics, anatomical stature estimate and the estimates obtained using the formulae presented in the article (1 to 5). Figure S1: Mean deviation of stature estimates from Anatomical Stature by formula presented in the study. Lowest deviations on the left and higher deviations on the right. [file AJPA-190-e70310-s001.pdf]
